# Parasitism with Protozoa and Monogeneans in Fish from the Natural Waters of Romania

**DOI:** 10.3390/microorganisms12081519

**Published:** 2024-07-24

**Authors:** Gheorghe Dărăbuș, Kristian Robert Ujvari, Mirela Imre

**Affiliations:** 1 Faculty of Veterinary Medicine, University of Life Sciences “King Mihai I” from Timisoara, 300645 Timișoara, Romania; gheorghedarabus@usvt.ro; 2 DSVSA Harghita, 530200 Miercurea Ciuc, Romania; ujvari.kristian@gmail.com

**Keywords:** protozoa, monogeneans, freshwater fish, prevalence

## Abstract

Parasitism by protozoa and monogenean flatworms in freshwater fish from Romania was studied by collecting and examining samples from two major river systems there: 183 fish from 17 species from the Olt River and its tributaries; and 155 fish from 16 species from the Mureș River and its tributary, Târnava Mare. The average rates of parasitism in the samples from the two rivers and their tributaries were as follows: *Ichthyiophthirius multifiliis* (2%), *Trichodina* spp. (21%), *Apiosoma* spp. (18%), *Mixobolus* spp. (8%), *Dactylogyrus* spp. (9%), and *Gyrodactylus* spp. (10%). The number of parasite species varied from one river to another. *I. multifiliis* was found in only 3 fish species, *Trichodina* spp. in 13 species, *Glosatella* spp. in 6 species, and *Mixobollus* spp., *Dactylogyrus* spp., and *Gyrodactylus* spp. in 7 different species each. The highest number of parasite species (six) were identified in the European chub (*Squalius cephalus*) and schneider (*Alburnoides bipunctatus*), which seem more susceptible to parasitic infections. The aquatic environment of these rivers may represent a source of parasites for fish from neighboring countries through which these rivers pass.

## 1. Introduction

Approximately 70% of diseases that affect fish are represented by parasitic diseases, and of these, 40% are of protozoan origin [1]. The economic damage caused by parasitic diseases in fish populations from both natural waters and fish farms can be significant [2,3,4,5]. Fish represent an important source of food for the human population, primarily in terms of protein [6]. Fish from natural sources of water are important not only as a food source, but also because they can represent an accidental source of parasites for fish farms [7]. Additionally, while fish grown in the natural environment have some resistance, those in farms do not have similar defense mechanisms, and their environmental conditions and way of feeding facilitate the rapid spread within populations and the development of massive infections [8].

Among the diseases with a negative impact on fish, the most important are ichthyophthyriasis, trichodinosis, Whirling disease, *Dactylogyrus* Gill Flukes Disease, and gyrodactylosis [9,10,11,12].

Ichthyophthyriasis is a ciliomatosis in freshwater fish, characterized by the development of whitish nodules on the skin and gills, with sometimes severe evolution and mortality, especially in fish farms [9,13]. Juveniles and young fish are more affected [14,15,16,17,18]. In natural waters, the evolution is less severe due to lower density [1,19]. Many freshwater fish species are affected, although not catfish (*Silurus glanis*) [20].

Trichodinosis is produced by a large number of mobile peritrichous ciliate species belonging to the family Urceolariidae. The parasites are located on the skin and gills of fish. Some authors consider them commensals rather than parasites [10]. However, of the many species, *Trichodina domerguei* appears to be more common and pathogenic [21]. All freshwater fish species are affected, but it can also parasitize marine fish [1,8].

Epistylids are sessile peritrich ciliate protozoa. Their pathogenicity is increased only in massive infections. Parasitism is encountered in numerous species of freshwater fish, with a higher frequency in cyprinids. Parasitism occurs in the warm season, especially in waters with excessive organic matter [1,22]. The parasites are localized on the gills and skin. In massive parasitism, parasite colonies give the appearance of “palisade gills” [8]. Cyprinids are frequently affected.

Whirling disease, originally considered to belong to the protozoa, is today classified in the Myxozoa phylum. Parasites are located on the gills, skin, spine, and central nervous system. Juveniles are more susceptible, while fish over 8 weeks of age do not develop the disease [1,23,24]. The main source of infection is water contaminated with spores, but oligochaetes of the genus Tubifex can also be a source of infection [25]. Salmonids are more susceptible, but the disease has also been reported in juvenile carp (*Cyprinus carpio*) [26].

Dactylogyrus Gill Flukes Disease is a monogenesis produced by worms of the genus Dactylogyrus. It is a cosmopolitan parasitosis, with localization in different areas of the gills, depending on the species [1]. The sources of infection are infected fish and the aquatic environment, the parasite eggs being highly resistant. The disease is triggered on hot summer days [8]. It is found in Cyprinidae, Percidae, Ictaluridae, and Anguillidae [1,27]

Gyrodactylosis is a gill and skin parasitosis [4]. Infection occurs through contact between infected and healthy fish [1,11,28]. The disease is widespread in Salmonidae, Cyprinidae, Actinopterygii, etc. In Romania, studies on protozoan and monogenic parasites in natural freshwaters are few and most of them refer to aquaculture fish [1,4,7,29] or are very old [30]. Given the small number of bibliographical references on the presence of protozoa and monogeneans, we undertook this scientific study. In addition, we relied on the fact that the prevalence of parasites can provide information about the health of the aquatic ecosystem [31].

This study aimed to (i) determine the prevalence and type of protozoa and monogenean parasites in fish from natural surface waters in Romania; (ii) identify the rivers where parasitism is more frequent, and (iii) investigate the differences in susceptibility to certain parasites for fish species inhabiting the water bodies.

## 2. Materials and Methods

### 2.1. Location

The study was carried out with fish material collected from several rivers and their tributaries in Romania. Thus, fish material was collected from several points of the Olt and Mures Rivers and their tributaries. The following streams flow into the Olt River: Madaras, Beta, Var, Aszo, Seghes, Fitod, Fisag, Nagyos, and Banya. The Homord River also flows into the Olt River, having its own two tributaries: Homorodul Mic and Homorodul Mare. The Tarnava Mare River flows into the Mures River. The selection of these rivers for the study was made taking into account the following aspects: they cross half of the territory of Romania, the situation of parasitic infections in fish was unknown, and these specific rivers, after water collection, pass through three other European countries namely Hungary, Serbia, and Bulgaria.

### 2.2. Materials

From the Olt River and its tributaries, 183 fish were collected and examined using various methods, while from the Mureș and Tarnava Mare Rivers, 145 fish were collected and examined.

The 183 fish from the Olt River and its tributaries belonged to 17 species: native trout (*Salmo trutta fario*) (n = 23), burbot (*Lota lota*) (n = 5), schneider (*Alburnoides bipunctatus*) (n = 25), the European chub (*Squalius cephalus*) (n = 20), rainbow trout (*Onchorhyncus mykiss*) (n = 1), brook trout (*Salvelinus fontinalis*) (n = 1), stone loach (*Barbatula barbatula*, sin. *Naemacheilus barbatulus*) (n = 12), spined loach (*Cobitis taenia*) (n = 4), gudgeon (*Gobio gobio*) (n = 25), common minnow (*Phoxinus phoxinus*) (n = 20), the Romanian barbel (*Barbus petenyi*) (n = 16), barbel (*Barbus barbus*) (n = 2), rutilus roach (*Rutilus rutilus*) (n = 10), gibel carp (*Carassius auratus gibelio*) (n = 1), perch (*Perca fluviatilis*) (n = 4), European bullhead (*Cottus gobio*) (n = 13), and common dace (*Leuciscus leuciscus*) (n = 1).

Fish from Mures and Tarnava Mare Rivers that could be collected and examined were classified into 16 species: the European chub (*Squalius cephalus*) (n = 39), common nase (*Chondrostoma nasus*) (n = 9), native trout (*Salmo trutta fario*) (n = 8), burbot (*Lota lota*) (n = 2), European grayling (*Thymallus thymallus*) (n = 5), gibel carp (*Carassius auratus gibelio*) (n = 1), common bleak (*Alburnus alburnus*) (n = 7), gudgeon (*Gobio gobio*) (n = 19), schneider (*Alburnoides bipunctatus*) (n = 28), rutilus roach (*Rutilus rutilus*) (n = 10), The Romanian barbel (*Barbus petenyi*) (n = 4), common barbel (*Barbus barbus*) (n = 2), common minnow (*Phoxinus phoxinus*) (n = 5), stone loach (*Noemacheilus barbatulus* syn. *Barbatula barbatula*) (n = 1), perch (*Perca fluviatilis*) (n = 3), and European bullhead (*Cottus gobio*) (n = 12).

### 2.3. Methods

The fish used for determining parasite species and the prevalence of parasitism were obtained randomly through fishing and the use of nets. The collection of fish from rivers was done from different pre-established points, chosen in a random way. Depending on the length of the river, 2 or 3 points were established at approximately equal distances for sample collection.

For the anatomopathological diagnosis, the methodology described by Cojocaru (2006) was applied. After an epidemiological investigation (water type, water color and turbidity, submerged and emerged vegetation, organic matter content, possible link with artificial fish breeding ponds, etc.), the macroscopic examination of the fish, i.e., external examination of the skin, fins, and gills, was carried out. Observations were made for modifications in conformation, excess mucus, lesions in fins, scales loss, possible congestion and hemorrhages, the presence of necrotic lesions and cysts, changes in skin color, spots, or punctuations [1]. To confirm the diagnosis, laboratory examinations were performed, including microscopic examination of scrapings from various body regions (gills, fins, skin). Mucus scraped with a scalpel from various body regions was deposited on a slide and examined under a microscope with 4×, 10×, 20×, 40×, and 100× objectives. The next step was the stereomicroscopic examination of the gills and fins. Then, the sedimentation method was performed after several successive washes of the gill arches and skin. The sediment obtained was examined stereomicroscopically and then microscopically. Additionally, anatomopathological examination of all internal organs was carried out [1].

For the diagnosis of ichthyophthyriosis, specific lesions were considered, namely the gray-white nodules present on the skin and gills, completed by microscopic examination of skin scrapings. The trophonts are large (up to 1 mm) and easy to identify, with *I. multifiliis* having cilia all over the body and a large reniform nucleus [1].

*Trichodina* species were identified by microscopic examination of the gills and skin. In the case of this pathogen, the goal was to examine the fish quickly because as time passes, the mucus layer increases, hindering the identification of parasites. The protozoan has the specific appearance of a turned saucer, with numerous cilia arranged marginally in a spiral. For *Trichodina*, the cilia spiral is 270° and the outer blade is flattened, with a central cone and a relatively well-developed inner radius. Depending on the species, they range in size from 26 µm to 75 µm [8,9].

To detect Epistylidae and Apiosoma (Glosatella), the fish were examined macroscopically. Infected fish have a thick layer of mucus on the body and wrinkled scales. Fish body and fin scrapings were performed and ciliated protozoa were identified microscopically. The Glosatella is cup-shaped and 23–43 µm long, and in the anterior part it has a cytostome surrounded by a crown of cilia and in the posterior part an adhesive disc [1,9]. Water samples were also taken to determine possible contamination with organic matter.

Mixosporidia were identified microscopically in the collected scrapings, mainly from the vertebral arches, or through the squash method. Microscopic examination of nodular lesions in the internal organs was also performed. The examination was conducted as soon as possible because of the possibility of structural changes in the protoplasm. Spores of the genus *Mixobolus* are spherical or ovoid, bivalve, with two polar capsules at one pole. Depending on the species, they range in size from 26 µm to 75 µm [8].

For the identification of parasitism with *Dactylogyrus* spp., microscopic examination of the gill scrapings was used in larger fish, and in smaller fish, successive washings of the gills were made, with examination of the sediment. The latter method avoids the detection of monogens, which are smaller and more difficult to detect in small fish. It was required to examine the fish as quickly as possible because of the alteration or mucus loading of the helminths, which does not allow the observation of their morphology [1,8].

Parasites of the genus *Gyrodactylus* are small and more difficult to detect after death, and therefore examination of the fish should be conducted as fast as possible. Scraps and sediments obtained by successive washings should be examined very carefully. It is best to send the fish to the laboratory alive, as monogeneans are destroyed by freezing. The genus *Gyrodactilus* differs from the genus *Dactylogyrus* in that instead of 14 hooks on the opisthaptor, they have 16 hooks; the prohaptor has no ocular pigmentation spots or ocelli; and they are viviparous [1,8].

## 3. Results

In the rivers and their tributaries in Romania examined in the study, parasitism was identified with *I. multiphiliis*, *Tricodina* spp., *Epistylidae, Mixobollus* spp., *Dactylogyrus* spp., and *Gyrodactylus* spp.

**Figure 1:**
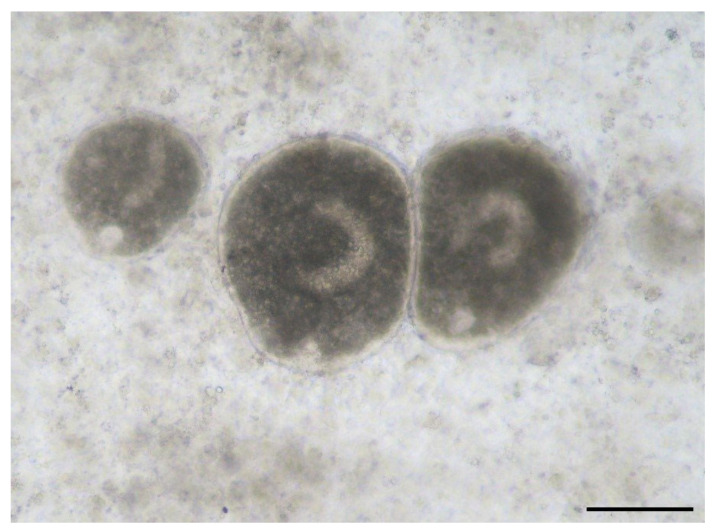
*Ichthyophthirius multifiliis* from carp gills (400× magnification) scale bar 30 μm.

Parasitism with *I. multifiliis* (Figure 1) was identified in only three fish species (Table 1), in the flowing waters downstream of the trout farms. This is explainable, as fish farms can be a source of parasites for the natural environment. However, no mortality has been reported in natural waters, probably due to the lack of high fish density. The specific ichthyophtyriasis lesions were found exclusively on gills. Only native trout, European chub, and schneider were positive for these parasites. Of the 13 fish examined, 5 (39%) were positive for parasites. Overall, considering that a total of 332 fish were examined, the overall prevalence of infection with *I. multifiliis* was 2%.

**Figure 2:**
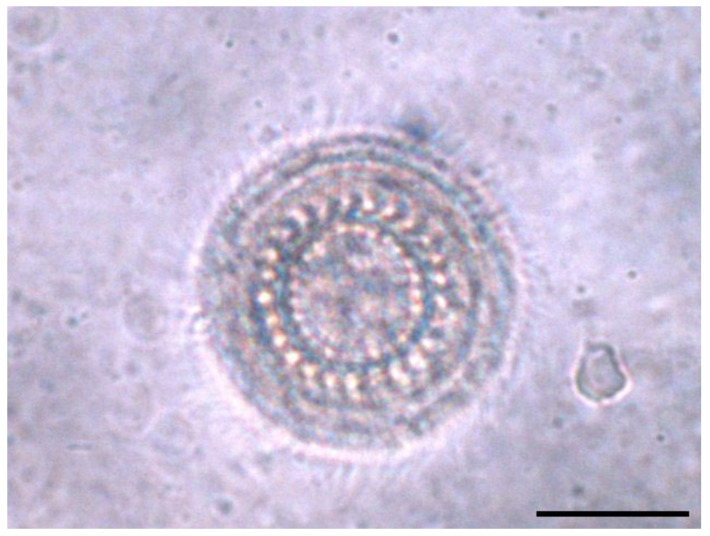
*Trichodina* spp. from carp gills (400× magnification) scale bar 30 μm.

Table 2 presents the fish species in which *Trichodina* spp. parasitism was identified (Figure 2). Thirteen fish species were found to be parasitized with this protozoan. The parasites were mainly identified on the gills, with fewer occurrences on the skin. No macroscopic morpho-pathological changes or behavioral disorders were observed. This suggests that in the natural environment, the intensity of parasitism is low, without obvious clinical manifestations. Considering only fish species in which *Trichodina* spp. parasitism was found, the prevalence of parasitism was 59%. However, based on the fact that there were also fish species in which parasites were not identified, the overall prevalence was 21%. Two of the species seem to have a high receptivity and appear to be more frequently parasitized: common minnow (*Phoxinus phoxinus*) and European chub (*Squalius cephalus*).

**Figure 3:**
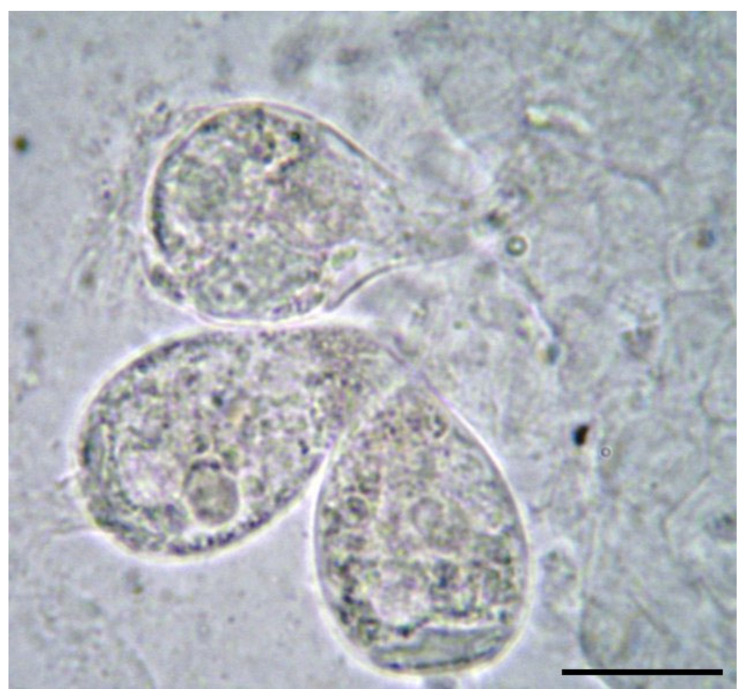
*Glosatella* spp. from carp skin (400× magnification) scale bar 30 μm.

Parasitism with *Epistylidae* was diagnosed in six species of fish (Table 3). Parasites from the genus *Glosatella* (Apiosoma) were identified (Figure 3). Affected fish were more easily captured, and had raised scales and excessive mucus on the skin. The favoring factor of infection was also identified, namely the excess of decomposed organic matter in the water due to rainwater washing down a garbage platform located upstream. In the six species of fish positive for this parasite, the prevalence was 100%. However, parasitism with *Glosatella* spp. reported among all fish captured from all natural waters examined in the study was 18%.

**Figure 4:**
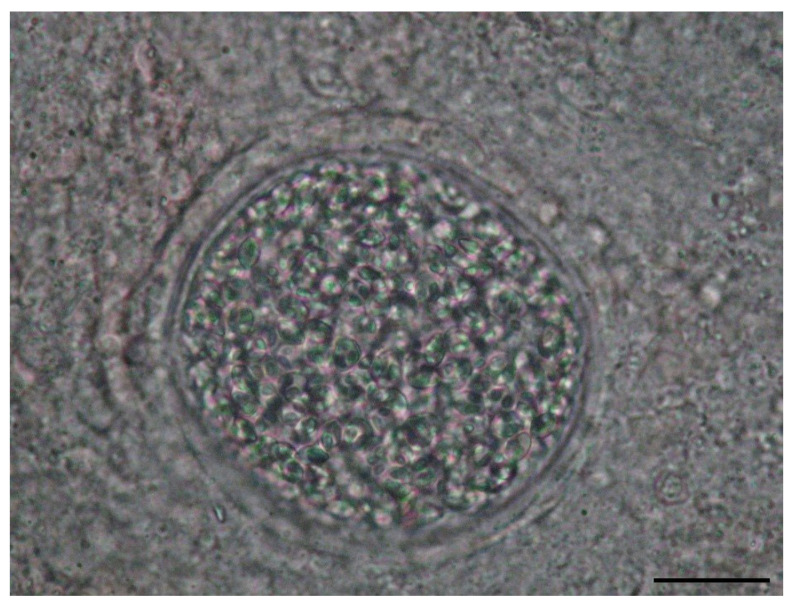
Cyst with myxospores from chub (*Squalius cephalus*) gill (400× magnification) scale bar 30 μm.

*Mixobolus* spp. (Figure 4), now classified within a separate phylum, Mixozoa, following investigations, was found in seven species of fish, in eight natural waters (Table 4). The localization was predominantly found on the gills. In cases of rare infections with renal localization, the parasites were not present on the gills. They probably belonged to different species. Parasitism seems to be more widespread in the European chub (*Squalius cephalus*) considering the large number of rivers where it was found. The prevalence, reported only for the seven fish species parasitized with *Mixobolus* spp., was 55%. However, reported among all fish examined, the prevalence of this parasitism was 8%.

**Figure 5:**
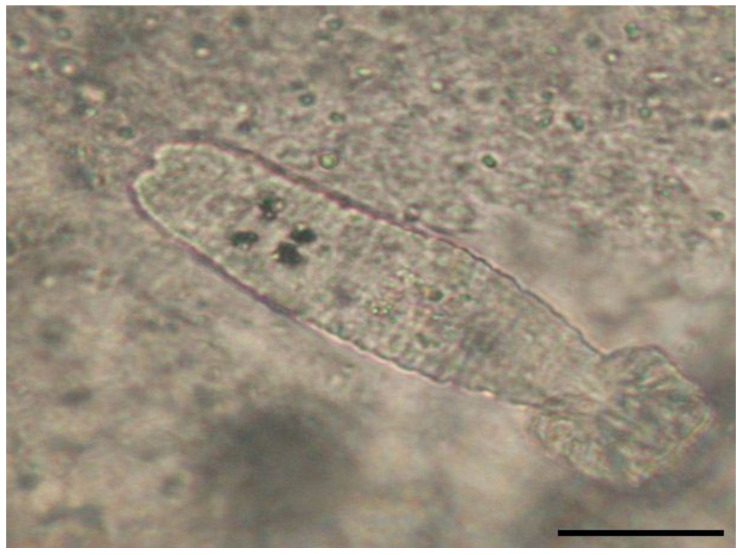
*Dactylogyrus* spp. from gills of one schneider (*Alburnoides bipunctatus*) (200× magnification) scale bar 50 μm.

Table 5 describes the parasitism status of *Dactylogyrus* spp. (Figure 5) in the examined flowing waters from Romania. Seven fish species (Table 5) were affected by this monogenean. It seems to be more widespread in the European chub (*Squalius cephalus*). The parasites were mainly located on the gills and less frequently on the skin and fins. Monogeneans are more difficult to detect in smaller fish. Performing gill scrapings before successive washings yielded inconclusive results. For the species where the parasite was identified, the prevalence was 31%. Overall, in the study, the prevalence of parasitism with *Dactylogyrus* was 9%.

**Figure 6:**
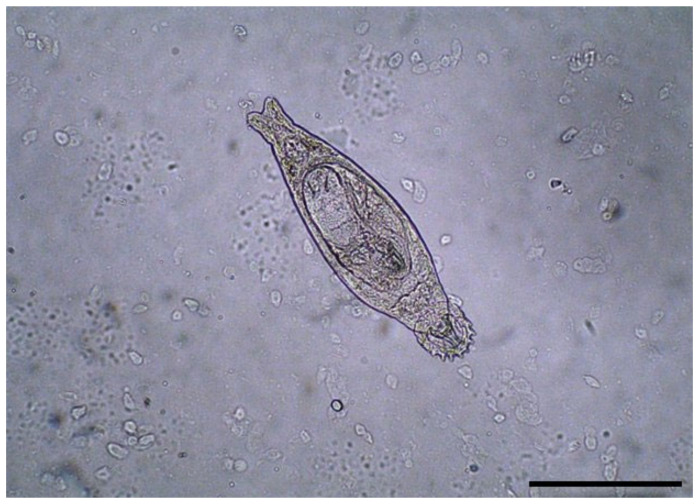
*Gyrodactyllus* spp. from brown trout (*Salmo trutta fario*) (100× magnification) scale bar 30 μm.

*Gyrodactylus* spp. in the seven parasitized fish species (Table 6) (Figure 6) was located equally on gills, skin, and fins. Erosions were reported in parasitized fish. It was found that for proper identification, due to the small size of parasites, it was advisable to bring the fish to the laboratory alive. In addition, the analysis of the sediment obtained from successive washings should be conducted with great care. In species where parasites were found, the prevalence was 45%. Overall, in fish collected from all rivers analyzed, the prevalence was 10%.

## 4. Discussion

In a recent study (2023) conducted in Romania, Darabus et al. [7] identified parasitism by *I. multifiliis* in fish from rivers, lakes, and fish farms. The presence of this parasite in our study is related to the source represented by the fish farms located close to the three rivers

In a similar study conducted in Tibet, with reference to *I. multifiliis* parasitism, the prevalence varied between 24% and 34%, depending on the fish species identified [9]. Another study, conducted in Egypt by Abd-ELrahman et al. [5], reports ectoparasitism with variable prevalence depending on the species parasitized, as follows: *Icthyophthirius multifiliis* (4%), *Trichodina* (6%), *Gyrodactylus* (5%), *Dactylogyrus* (4%), and *Cichlidogyrus* (22%) [5]. Similarly, in Iran, in *Cyprinus carpio*, parasitism with *Dactylogyrus extensus* (29%), *Dactylogyrus anchoratus* (2%), *Ichthyophthirius multifiliis* (10%), *Trichodina nigra* (7%), *Capillaria* spp. (5%), *Procaecum* spp. (2%), *Argulus foliaceus* (2%), and *Lernaea cyprinacea* (4%) was found [13].

A study carried out in Brazil on several species of juvenile fish collected from a fish farm reveals the presence of 10 species of parasites, including *Apiosoma* spp., *Epistylis* spp., *Ichthyobodo* spp., trichodinids, monogeneans, and *Ichthyophthirius multifiliis* [32].

In the freshwaters of the Nile in Assiut Governorate, Upper Egypt, a prevalence of parasitism with *Mixobolus* spp. of 2% was identified in 300 tilapia (*Oreochromis niloticus*) samples [5].

Fariya et al. (2022) identified parasitism with *Myxobolus grassi* sp. nov. in grass carp (*Ctenopharyngodon idella*), a freshwater fish. The prevalence was 4%, with the parasite being localized on the gills as the primary site and in the liver as the secondary site of infection [33]. A study in Beni-Suef, Egypt, on 77 Nile tilapia (*Oreochromis niloticus*), determined a very high prevalence of myxosporean infection. High numbers of free spores were found in the kidneys and spleen. The prevalence was 52% (40/77) for *Myxobolus brachysporus* and 26% (20/77) for *Myxobolus israelensis* [34].

In a study carried out in Romania, two species of *Gyrodactylus* were found, *G. salaris* and *G. truttae*. *Gyrodactylus salaris* was found in rainbow trout, brown trout, and brook trout in 8 of the 12 farms examined. The prevalence and intensity of infections were low [29].

In Brazil, Afiyah et al. (2023) identified in common carp (*Ciprinus carpiao*) the following parasite species with varying prevalence: *Trichodina* sp. (100%), *Dactylogyrus* sp. (95%), *Gyrodactylus* sp. (47%), *Myxobolus* sp. (32%), *Thelohanellus* sp. (13%), and *Ichthyophthirius* (7%) [35].

Molnár et al. (2010), in Hungarian freshwaters, identified eight species of *Mixobolus* in rutilus roach (*Rutilus rutilus*) [36]. Also, in another study, myxosporean infections were found in bleak (*Alburnus alburnus*) in Danube waters [37]. In a summary study conducted in Serbia by Djikanović, et al. [38] on parasitism in fish from the Danube basin, protozoan parasites were found: *Mixobollus* spp., *I. multifiliis*, *Trichodina* spp., *Chilodonella* spp., *Apiosoma* spp., *Tricodonella*, and *Balantidium* spp.; and among monogeneans: *Gyrodactylus derjavini* and *Urocleidus similis*.

If we analyze the number of fish species affected by protozoan and monogenean parasites, we find that there are significant differences among the parasite species. Thus, *I. multifiliis* was found in only 3 fish species, *Trichodina* spp. in 13 species, *Glosatella* spp. in 6 species, and *Mixobollus* spp., *Dactylogyrus* spp., and *Gyrodactylus* spp. in 7 different species each.

Fish species collected from the studied rivers were parasitized with a variable number of parasite species (Table 1, Table 2, Table 3, Table 4, Table 5 and Table 6). The most parasitized species, six each, were identified in the European chub (*Squalius cephalus*) and schneider (*Alburnoides bipunctatus*) fish species, followed by native trout (*Salmo trutta fario*) with five parasitized species, and gudgeon (*Gobio gobio*) and common minnow (*Phoxinus phoxinus*) with four parasitized species each.

## 5. Conclusions

Parasitism with protozoa and monogeneans in fish from natural waters in Romania is different and found in many fish species. Parasitism with *I. multifiliis*, quite widespread in fish farms [7], is rare in fish species from naturally flowing waters.

*Trichodina* parasitism is most spread in fish species originating from flowing waters in Romania (21%). Two of the fish species in rivers seem to be more frequently positive for parasites: common minnow (*Phoxinus phoxinus*) and European chub (*Squalius cephalus*).

Parasitism with *Glosatella* spp. quite high (18%), and diagnosed in six fish species, appears to be due to excess organic matter in the waters where parasitism was identified.

Parasitism with *Mixobollus* spp. was identified in seven fish species, with a prevalence of 8%. *Mixobollus* spp. was found mostly in the European chub (*Squalius cephalus*), which appears to be a more susceptible species.

Parasitism rates with monogeneans in the flowing waters of Romania show close prevalence within the two identified species: *Dactylogyrus* spp. (9%) and *Gyrodactylus* spp. (10%).

*I. multifiliis* was identified in only three fish species, while parasitism with *Trichodina* affected 13 fish species. The affected fish species with the highest number of parasite species were the European chub (*Squalius cephalus*) and schneider (*Alburnoides bipunctatus*).

The study opens the way for further research to elucidate the factors that contribute to differences in parasitism found between the different fish species.

## Figures and Tables

**Table 1:** Parasitism with *I. multifiliis* in Romanian rivers.

Fish Species Scientific Name	Fish Species Common Name	River and Tributaries	Positive Samples	/Total Number of Samples
*Salmo trutta fario*	Native trout	Mădăraș	1	4
*Squalius cephalus*	European chub	Homorodul Mare	2	4
*Alburnoides bipunctatus*	Schneider	Tarnava Mare	2	5

**Table 2:** Parasitism with *Trichodina* spp. in Romanian rivers.

Fish Species Scientific Name	Fish Species Common Name	River and Tributaries (Positive/Total Number)
*Ciprinus carpio*	Common carp	Olt (2/2)
*Carassius auratus gibelio*	Gibel carp	Fitod (1/1); Olt (5/5)
*Salmo trutta fario*	Native trout	Mădăraș (1/4), Fișag (1/1); Beta (1/4); Var (1/5)
*Thymallus thymallus*	European grayling	Mureș (1/1)
*Phoxinus phoxinus*	Common minnow	Beta (1/1), Fișag (2/2); Fitod (1/1); Aszo (3/3); Mădăraș (2/4)
*Squalius cephalus*	European chub	Fitod (1/2); Fișag (2/2); Mureș (6/18); Homorodul mic (1/2); Olt (6/10)
*Alburnoides bipunctatus*	Schneider	Olt (2/4); Homorodul mic (4/8)
*Barbus meridionalis petenyi*	Romanian barbel	Mureș (2/2)
*Gobio gobio*	Gudgeon	Fișag (2/3); Fitod (2/3); Homorodul mic (3/5); Mădăraș (1/1)
*Rutilus rutilus*	Rutilus roach	Olt (2/10)
*Noemacheilus barbatulus*	Stone loach	Beta (1/1); Fișag (1/1); Fitod (2/3); Homorodul mic (3/3)
*Cobitis taenia*	Spined loach	Fișag (2/2); Aszo (2/2)
*Lota lota*	Burbot	Beta (3/3); Mureș (1/1)

**Table 3:** Parasitism with *Epistylidae* in Romanian rivers.

Fish Species Scientific Name	Fish Species Common Name	River and Tributaries (Positive/Total Number)
*Alburnoides bipunctatus*	Schneider	Olt (4/4); Homorodul mic (8/8); Fișag (10/10)
*Gobio gobio*	Gudgeon	Fișag (3/3); Fitod (3/3); Banya (1/1)
*Carasssius auratus gibelio*	Gibel carp	Fitod (1/1)
*Squalius cephalus*	European chub	Fișag (2/2); Olt (10/10)
*Salmo trutta fario*	Native trout	Mădăraș (4/4); Olt (3/3); Târnava mare (3/3); Aszo (4/4)
*Phoxinus phoxinus*	Common minnow	Aszo (3/3)

**Table 4:** Parasitism with *Mixobollus* spp. in Romanian rivers.

Fish Species Scientific Name	Fish Species Common Name	River and Tributaries (Positive/Total Number)
*Phoxinus phoxinus*	Common minnow	Aszo (1/3)
*Squalius cephalus*	European chub	Fișag (1/2); Mureș (2/9); Fitod (1/2); Târnava mare (2/2); Olt (10/20); Homorodul Mic (1/2); Homorodul Mare (1/1);
*Alburnus alburnus*	Common bleak	Mureș (1/1)
*Alburnoides bipunctatus*	Schneider	Olt (2/4); Homorodul mic (1/4)
*Chondrostoma nasus*	Common nase	Mureș (2/2)
*Rutilus rutilus*	Rutilus roach	Mureș (1/4)
*Lota lota*	Burbot	Beta (1/3)

**Table 5:** Parasitism with *Dactylogyrus* spp. in Romanian rivers.

Fish Species Scientific Name	Fish Species Common Name	River and Tributaries (Positive/Total Number)
*Salmo trutta fario*	Native trout	Aszo (4/4)
*Squalius cephalus*	European chub	Mureș (2/18); Târnava mare (1/12); Homorodul Mic (1/2); Olt (1/10)
*Alburnus alburnus*	Common bleak	Târnava mare (2/2)
*Alburnoides bipunctatus*	Schneider	Olt (1/4); Homorodul mic (4/8); Mureș (5/15)
*Barbus petenyi*	Romanian barbel	Homorodul mic (2/4); Târnava mare (2/2)
*Gobio gobio*	Gudgeon	Fișag (2/3)
*Rutilus rutilus*	Rutilus roach	Mureș (2/10)

**Table 6:** Parasitism with *Gyrodactylus* spp. in Romanian rivers.

Fish Species Scientific Name	Fish Species Common Name	River and Tributaries (Positive/Total Number)
*Salmo trutta fario*	Native trout	Aszo (1/4); Beta (1/4); Mădăraș (3/4); Olt (3/3)
*Phoxinus phoxinus*	Common minnow	Aszo (2/3); Mădăraș (1/4); Fișag (1/2)
*Squalius cephalus*	European chub	Fișag (1/2); Olt (4/10)
*Alburnoides bipunctatus*	Schneider	Olt (3/4); Homorodul mic (1/8); Târnava mare (1/13)
*Barbus petenyi*	Romanian barbel	Var (2/2); Fișag (5/5)
*Gobio gobio*	Gudgeon	Mădăraș (1/1)
*Noemacheilus barbatulus*	Stone loach	Fișag (1/1); Fitod (2/3)

## Data Availability

The original contributions presented in the study are included in the article, further inquiries can be directed to the corresponding author.
